# Circulating Tumor Cells Were Associated with the Number of T Lymphocyte Subsets and NK Cells in Peripheral Blood in Advanced Non-Small-Cell Lung Cancer

**DOI:** 10.1155/2017/5727815

**Published:** 2017-12-24

**Authors:** Liang Ye, Fang Zhang, Huijuan Li, Linfei Yang, Tangfeng Lv, Wei Gu, Yong Song

**Affiliations:** ^1^Department of Respiratory Medicine, Nanjing First Hospital, Nanjing Medical University, Nanjing 210006, China; ^2^Department of Respiratory Medicine, Jinling Clinical Medical College of Nanjing Medical University, Nanjing 210006, China; ^3^Department of Respiratory Medicine, Jinling Hospital, Nanjing University School of Medicine, Nanjing 210006, China

## Abstract

In this study, we aim to investigate the correlation between circulating tumor cells (CTCs) and the T lymphocyte subsets and NK cells in peripheral blood in non-small-cell lung cancer (NSCLC). The peripheral blood CTCs were determined by SET-iFISH. Flow cytometry was used to determine the distribution of T lymphocyte subsets and NK cells. Forty-one (49%) patients showed positivity for CTCs. Logistic regression analysis revealed CTC number was negatively correlated with the ratio of CD3+, CD4+, CD4+/CD8+, and NK % in patients at stage IV, while in a positive correlation was noticed between CTC number and regulatory T cell (Tregs) ratio in these patients. Multivariate analysis was performed in combination with the clinical-pathological materials to identify the risk factors for CTC positivity. Differentiation, NSCLC stage, percentages of CD3+CD4+ cells, Tregs, and NK cells were the independent risk factors for CTCs. CTCs were associated with the decrease of immune surveillance in the peripheral blood in NSCLC patients. The decrease of immune surveillance contributed to the escape of CTCs from the killing effects of the immunocytes, as well as the formation of metastasized lesions in the target organs.

## 1. Introduction

Lung cancer is the leading cause of cancer-related mortality worldwide. Most of the lung cancer patients usually die from recurrence and distal metastasis [1–3]. Nowadays, the management of lung cancer is still a challenge. Blood dissemination is the major cause for metastasis of lung cancer, through which distal metastasis is achieved with blood circulation [4, 5]. Circulating tumor cells (CTCs) represent a heterogeneous population of malignant cells that disseminate into the blood circulation, escaping from the immune surveillance in the presence of various cytokines. Under certain conditions, these cells could enter the target organs and contribute to the metastasis of cancer cells. On this basis, CTCs are considered as a prognostic factor in primary and metastatic cancers [6, 7].

Cancer cells have been detected in the peripheral blood in the patients with solid tumor, and the CTCs are considered as the origin for the metastasis and recurrence. Besides, CTC enumeration offers potential utility as a prognostic biomarker and predictor, and it may fulfill the criteria for a surrogate response biomarker [8, 9].

In the past decades, the immune system was reported to inhibit the progression of cancer cells [10–12]. In healthy individuals, a large number of immunocytes are detected in the peripheral blood, including T lymphocytes, NK cells and B lymphocytes, which play crucial roles in the immune surveillance, immunosuppression and killing effects. Such process is considered to be highly related to the CTCs [13–15]. For example, cytotoxic sinusoidal lymphocytes of liver transplant were reported to be effective against CTCs [16]. Cancer patients were usually in an immunocompromised state, together with a decrease of immunocytes in the peripheral blood. On this basis, CTCs may escape the immune response and access to the target organ through blood circulation, which contributed to the metastasis.

To date, rare studies have been focusing on the correlation between CTCs and the distribution of immunocytes in peripheral blood. In a previous study, Mego et al. [17] reported that CTCs were associated with the defect of adaptive immunity in breast cancer patients. In this study, we aim to investigate the correlation between CTCs and T lymphocyte subsets in the peripheral blood in non-small cell lung cancer (NSCLC) patients.

## 2. Materials and Methods

### 2.1. Patients

Eighty-three late-stage primary NSCLC patients admitted to our hospital from November 2013 to January 2015 were included in this study. NSCLC was confirmed using pathological analysis, and the staging of the late-stage NSCLC (IIIa, IIIb, and IV) was carried out according to the seventh edition of the American Joint Committee on Cancer (AJCC) staging manual. Clinical data including age, ethnicity, histological subtype, smoking status, and sites of metastasis were collected. The inclusion criteria were as follows: those with a WHO Performance Status of 0–2; those who received no radiotherapy or chemotherapy before; and those with an expected survival of >3 months. The exclusion criteria were as follows: those with a history of malignancy within 5 years and those with severe disorders or complications (e.g., heart failure). Thirty-five healthy individuals received physical examinations in our hospital served as healthy control. Written informed consent was obtained from each subject. The study protocols were approved by the Ethics Committee of Nanjing First Hospital, Nanjing Medical University.

### 2.2. Detection of CTCs by SET-iFISH

**Figure 1:**
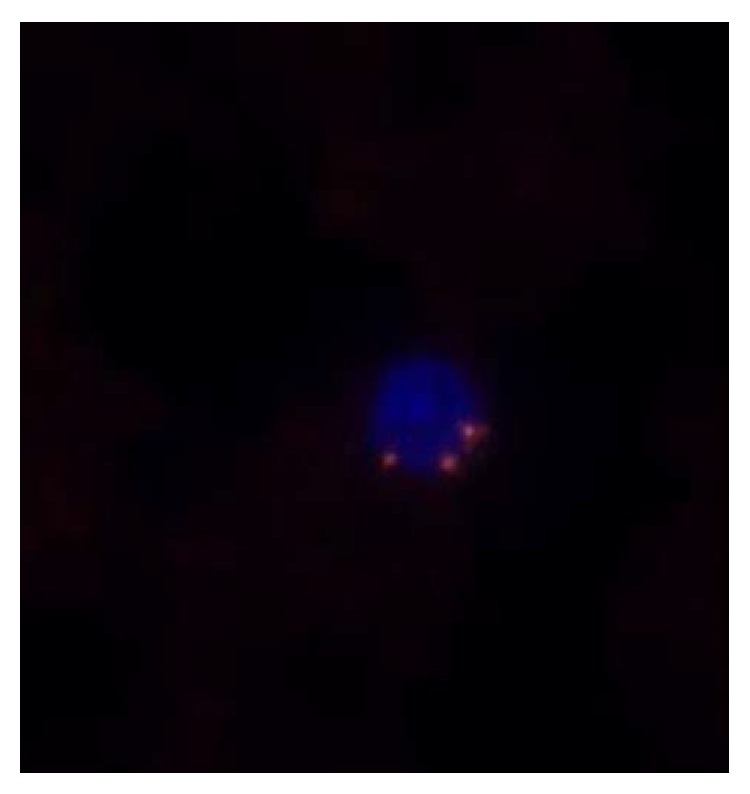
Immunostaining of a single lung cancer CTC isolated from patient peripheral blood. The CTCs were observed through combining CD45, DAPI, and fluorescence in situ hybridization with the centromere of chromosome 8 (CEP8). CD45-DAPI+CEP8>2 was considered as CTC.

The enrichment of CTCs was carried out according to the previous description (18, 19) with slight modifications. Briefly, peripheral blood (4 ml) was treated using lysis of red blood cells. Afterwards, the pellets were resuspended in PBS buffer followed by incubating with anti-CD45 monoclonal antibody-coated magnetic beads for 30 min. The mixture was separated by magnetic beads using a magnetic stand (Promega, Madison, WI, USA). Enriched lung cancer CTCs were identified using CD45-FISH as previously described [16]. The CEP8 probe and specimen were hybridized in DAKO at 37°C for 20 min and then washed in 50% formamide at 43°C for 15 min. Finally, the specimens were washed with 0.2% BSA, followed by incubating with the CD45 mixture/2% BSA conjugated to Alexa Fluor 594 (Invitrogen) for 1 h. The specimens were then stained with DAPI, and the fixed sample should be observed entirely under a microscope (Nikon). Positive CTCs were defined as hyperdiploid CEP8+/DAPI+/CD45− (Figure 1). The CTC count of >2 in 4 mL blood was considered positive.

### 2.3. Analysis of T Lymphocyte Subsets in Peripheral Blood

Peripheral blood mononuclear cells (PBMCs) were isolated using the Ficoll–Paque (Pharmacia) density-gradient centrifugation. After blocking with FcR, PBMCs were incubated with diluted antibodies for phenotyping and then analyzed using flow cytometry (FC-500, Beckman Coulter, FL, USA). Data analysis was conducted using FlowJo software (version 7.6.2, Tree Star, Ashland, OR, USA). Circulating T lymphocyte subgroup and NK cells were identified by multiparameter flow cytometry according to the previous description [18, 19]. T lymphocyte subsets were identifying CD4-FITC/CD8-PE/CD3-PC5, and NK cell was identifying with CD3-FICT/CD(16+56)-PE (BD Biosciences, CA, USA). The phenotype of NK cells was CD3-CD16+CD56+. Four-color flow cytometry (FC-500, Beckman Coulter, FL, USA) was performed to determine the phenotypes of T regulatory cells (Tregs) using the CD3-PC5, CD4-PE, CD25-FITC (BD Biosciences, CA, USA), and CD127-PC7 antibodies (BioLegend, San Diego, CA, USA). Tregs were defined as CD3+CD4+CD25+CD127− cells.

### 2.4. Statistical Analysis

The data are expressed as means ± standard error of the mean (SEM) unless specified. The association between CTCs and individual clinical characteristics (e.g., stage, PS, histology, smoking, and sites of metastasis) was compared by Fisher's exact test. Normality of distribution was tested by the Kolmogorov-Smirnov test. If data were normally distributed, analysis of variance was used for the intergroup comparison. For data with not normally distributed, the equivalent nonparametric tests were performed and data were expressed as median (interquartile range). A least significant difference (LSD) test was used for paired comparison between two groups in presence of statistical significance after comparison among multiple groups. Student's *t*-test was used to compare the parameters between immune cells and CTC status. Univariate analysis was depicted using Pearson's coefficient. A multilinear regression analysis was performed with immune cells, tumor grade (1 and 2 versus 3), and tumor stage (stages IIIa and IIIb IV versus stage IV) as the independent variables and CTCs as the dependent variable. *P* < 0.05 was considered statistically significant.

## 3. Results

### 3.1. Demographic Information

Eighty-three late-stage NSCLC patients were included in this study, among whom 14 were of IIIa stage, 17 of IIIb stage, and 52 of IV stage, respectively. Compared with the healthy individuals, no statistical difference was noticed in the age and sex ratio in the late-stage NSCLC patients (*P* > 0.05, Table 1).

### 3.2. Association between CTCs and NSCLC

Forty-one (49%) patients showed positivity for CTC. No correlation was noticed between CTC enumeration and the histological features (*P* > 0.05, Table 1). The CTC number showed no statistical difference in patients aged ≤ 60 yrs compared with those aged > 60 yrs (*P* > 0.05). Whereas, higher CTC number was correlated with the pathological stages (*P* < 0.05). To be exact, the number of CTC in the patients of the IV stage was significantly higher than that of the counterparts of IIIa and IIIb, respectively. Besides, the CTC-positive rate was statistically correlated with the differentiation of cancer cells (*P* < 0.05).

### 3.3. Alternations of T Lymphocyte Subsets and NK Cells in Late-Stage NSCLC Patients

Compared with the healthy control, the ratio of CD3+, CD3+CD4+, and NK cells was decreased in the T lymphocytes in the peripheral blood of patients with late-stage NSCLC (*P* < 0.05, Table 2). Besides, the ratio of Tregs was significantly elevated in the late-stage NSCLC patients compared with the healthy control (*P* < 0.05). Compared with the IIIa or IIIb NSCLC patients, a significant decrease was observed in the ratio of CD3+, CD3+CD4+, CD4+/CD8+, and NK cells in those with at the IV stage. Besides, significant elevation was observed in the Tregs ratio in the patients at IV stage compared with those at IIIa or IIIb. No statistical difference was noticed in the T lymphocyte subsets between the NSCLC patients at IIIa and IIIb stages (*P* > 0.05).

### 3.4. Correlation between CTCs and T Lymphocyte Subsets and NK Cells

Univariate analysis showed the percentages of CD3+, CD4+, and NK cells in CTC-positive patients were significantly decreased compared with those of the CTC-negative patients. In contrast, the ratio of Tregs in CTC-positive patients was obviously higher than that of the CTC-negative patients (*P* < 0.05, Table 3). Logistic regression analysis revealed CTC number was negatively correlated with the ratio of CD3+, CD4+, CD4+/CD8+, and NK% in patients at stage IV, while in a positive correlation was noticed between CTC number and Tregs ratio in these patients (Table 4). Furthermore, multivariate analysis was performed in combination with the clinical-pathological materials to identify the risk factors for CTC positivity. As revealed in Table 5, differentiation, NSCLC stage, and percentages of CD3+CD4+ cells, Tregs, and NK cells were the independent risk factors for CTCs.

## 4. Discussion

In line with the previous study [9], we testified the presence of CTCs in the peripheral blood in the late-stage NSCLC patients. For the first time, our study revealed the correlation between lymphocyte subsets and CTCs in the peripheral blood of NSCLC patients, which implied a close relationship between the decrease of peripheral immune surveillance and CTCs. Such process was featured by a decreased ratio of NK cells, CD3+, CT4+, and T cells, as well as elevation of the Treg ratio. The immune disorder contributed to the dissemination of cancer cells into the circulation that escaped the killing effects of the immunocytes and formation of metastasis after migrating to the target organs, which finally resulted in disease progression and a poor survival rate.

Up to now, several methods have confirmed the presence of CTCs in NSCLC patients, especially the late-stage patients. Besides, a correlation was established between the CTC number and the clinical staging of the disease, which was considered to play important roles in the early screening, efficiency monitoring, and prognosis evaluation, as well as preparation of regimen for the individual therapy [20–23]. In this study, we determined the CTC number in peripheral blood using the SET-iFISH in 83 cases with late-stage NSCLC, among which 41 (49%) showed positivity for CTC screening. Besides, the number of CTCs in the patients at stage IV was obviously higher than those at stage III. In line with the previous study [24], no CTCs were detected in the peripheral blood of the healthy individuals. Meanwhile, we also analyzed the correlation between CTC-positive rate and the clinical-pathological features of the late-stage NSCLC patients, which demonstrated clinical staging, differentiation of cancer cells, and distal metastasis were the risk factors for CTC positivity.

As is known to all, abundant immunocytes were presented in the peripheral blood, including T lymphocytes, B lymphocytes, and NK cells. These cells could kill the cancer cells that desquamated into the peripheral blood [25–28]. CTCs had been detected in the peripheral blood in patients with lung cancer; however, the exact mechanism of how CTCs escape from the killing effects of the immunocytes is still not well defined.

Increasing evidence shows the immune status is closely related to the pathogenesis and development of cancer in hosts [29–31]. Under normal conditions, the interaction between the T cell subsets is crucial for the immune function in the individuals. Whereas, in cases of aberrant alternations in the quantity and function of the T cell subsets, the cancer cells may escape from the immune attack in the presence of immune dysfunction and pathological changes. To our best knowledge, T cell subsets consist of various subsets with different functions, among which CD3+ cells are defined as the total T lymphocytes representing the whole immune status including CD4+ and CD8+ cells. NK cells play important roles in the antitumor immunity and inhibition of the dissemination of malignant cancer cells. These cells could kill the cancer cells through secreting cytotoxic cytokines in the absence of sensitization, which contributed to the prevention of cancer invasion and metastasis and finally prevented the early dissemination of cancer cells. Tregs, as a T lymphocyte subset that could negatively modulate the immune function, could inhibit the development and differentiation of the effector cells that could recognize the cancer cells. On this basis, Treg was considered to closely participate in the immunological tolerance of cancer cells. Recently, extensive studies have indicated elevation of Tregs in the tumor microenvironment or peripheral blood as a predictor for progression and poor prognosis [28, 32]. It has been well acknowledged that cancer patients may present aberrant changes in the T lymphocyte subsets in the peripheral blood, which was reported to be related to the immune response imbalance due to imbalance of CD3+, CD4+, and CD8+ cells upon onset of malignant lesions, particularly the disturbance of the CD4+/CD8+ balance [28, 33]. In this study, flow cytometry was used to analyze the T lymphocyte subsets and NK cells in the peripheral blood in late-stage NSCLC patients. Our results indicated that the number of CD3+ and CD3+CD4+cells in the total T lymphocytes was significantly decreased in the peripheral blood of IV stage NSCLC patients compared with the healthy control. Additionally, the ratio of Tregs in the patients was obviously elevated compared with the healthy control. Taken together, we concluded that immune function disorder may present in the late-stage NSCLC patients, which was consistent with the previous descriptions [28, 32–34]. In particular, the decrease of CD3+, CD3+CD4+, and NK cell number and elevation of Tregs in the patients at stage IV was severe compared with these at stage III, which implied that the immune function disorder may be related to the clinical staging and distal metastasis. These findings suggested the presence of immunosuppression in the NSCLC patients, together with a decrease in the recognition and killing efficiency by the immune system towards the cancer cells, which resulted in the growth and metastasis of cancer cells. On this basis, it is reasonable to conclude that the immune function in the late-stage NSCLC patients is severely damaged, which hampers the recognition and killing of cancer cells by the host and triggers the extensive dissemination of cancer cells accordingly.

In order to investigate the correlation between the CTC number and the immunocyte distribution or clinical-pathological features, correlation analyses were performed between CTC number and distribution of immunocytes. Our data showed CTC number was positively correlated with the ratio of CD3+, CD3+CD4+, CD4+/CD8+, and percentage of NK cells, while it was negatively correlated with the ratio of Tregs. Multiple regression analysis showed CTC positive rate was correlated with the staging and distal metastasis, as well as immunocyte status in the peripheral blood. Besides, several factors have been identified as the risk factors for CTC positivity, including low CD3+ percentage, CD3+CD4+, and NK cells, as well as high Treg ratio. This indicated that the number of effector T cells showed persistent decrease with the disease progression, while the number of immunosuppressive cells was gradually increased. The balance of T lymphocyte subsets was disrupted with the disease progression together with inhibition of immune function, which resulted in the escape of cancer cells disseminated in the peripheral blood from the immune surveillance. Therefore, our results confirmed the CTCs were closely related to the aberrant distribution of T lymphocyte subsets in the peripheral blood in late-stage NSCLC patients.

In conclusion, we firstly investigated the correlation between CTCs and the aberrant distribution of T lymphocyte subsets in the peripheral blood in late-stage NSCLC patients. Multivariate analysis indicated differentiation, NSCLC stage, and percentages of CD3+CD4+ cells, Tregs, and NK cells were the independent risk factors for CTCs. In the future, further prospective studies are needed to investigate the correlation between CTCs and the immunocyte number and function.

## Figures and Tables

**Table 1:** Relationship between the presence of circulating tumor cells (CTCs) and the clinical features of non-small-cell lung cancer.

Clinical features	Number of cases	CTC-positive (%)	CTC-negative (%)	*χ*2	*P* values
Age					
≤60 years old	23	15 (65.2)	8 (34.8)	3.186	0.18
>60 years	60	26 (43.3)	34 (56.7)		
Gender					
Male	48	24 (50.0)	24 (50.0)	0.017	0.90
Female	35	17 (48.6)	18 (51.4)		
Smoking					
Yes	36	16 (61.5)	20 (38.5)	0.474	0.49
No	47	25 (53.2)	22 (46.8)		
Histological features					
Adenocarcinoma	35	17 (48.6)	18 (51.4)	0.184	0.91
Squamous cell carcinoma	41	20 (48.8)	21 (51.2)		
Others	7	4 (57.1)	3 (42.9)		
Tumor grade					
High differentiation	17	5 (29.4)	12 (70.6)	11.183	0.04
Moderate differentiation	36	14 (38.9)	22 (61.1)		
Poor differentiation	30	22 (73.3)	8 (26.7)		
Pathological stage					
IIIA	14	2 (14.3)	12 (85.7)	18.129	0.00
IIIB	17	4 (23.5)	13 (76.5)		
IV	52	35 (49.4)	17 (50.6)		

**Table 2:** Association between NSCLC and different subpopulations of T cells (mean ± SD).

Variables (%)	Control *n* = 35	Stage IIIa *n* = 14	Stage IIIb *n* = 17	Stage IV *n* = 52	*F* value	*P* value
CD3+	71.7 ± 2.5	69.8 ± 1.6^∗^	69.7 ± 1.8^∗^	65.7 ± 2.7^∗^^†§^	46.7	0.00
CD4+	39.4 ± 0.9	37.0 ± 1.2^∗^	36.9 ± 0.9^∗^^†^	31.7 ± 3.5^∗^^§^	74.4	0.00
CD8+	29.2 ± 0.7	29.3 ± 0.9	29.6 ± .9	29.5 ± 1.1	0.79	0.61
CD4+/CD8+	1.4 ± 0.1	1.3 ± 0.1^∗^	1.3 ± 0.1^∗^	1.1 ± 0.1^∗^^†§^	106.5	0.00
NK	18.1 ± 1.2	15.8 ± 0.9^∗^	15.7 ± 0.8^∗^	14.6 ± 1.2^∗^^†§^	70.4	0.00
Treg cell	5.4 ± 0.4	7.4 ± 0.5	8.0 ± 1.0	8.9 ± 1.6	63.2	0.01

^∗^ *P* < 0.05, versus control; ^†^*P* < 0.05, versus stage IIIa; ^§^*P* < 0.05, versus stage IIIb.

**Table 3:** Univariate analysis between CTC count (per 4.5 mL peripheral blood) and different subpopulations of T cells.

Variables	CTC-positive	CTC-negative	*T* value	*P* value
CD3+	64.9 ± 2.4	69.2 ± 2.1	8.78	0.041
CD4+	30.3 ± 2.8	36.5 ± 1.7	12.40	0.003
CD8+	29.2 ± 0.9	29.7 ± 1.0	2.29	0.173
CD4+/CD8+	1.0 ± 0.1	1.2 ± 0.8	11.07	0.235
NK	14.2 ± 0.9	15.7 ± 0.9	7.13	0.026
Treg cell	9.4 ± 1.2	7.6 ± 1.0	−7.07	0.004

**Table 4:** Correlation between CTC count and different subpopulations of T cells.

Parameters	Correlation coefficient	*P* value
CD3+	−0.735	<0.001
CD4+	−0.716	<0.001
CD8+	1.002	0.075
CD4+/CD8+	−0.943	0.061
NK	−0.648	<0.001
Treg cell	0.631	<0.001

**Table 5:** Multivariate logistic regression of the CTC positive model.

Variables	Odds ratio	95% CI low	95% CI upper	*P* value
% CD3+	0.65	0.43	0.98	0.031
% CD3+CD4+	0.96	0.75	1.03	0.043
% Treg	2.36	1.21	6.34	0.027
% NK	0.86	0.75	0.95	0.012
Grade	9.73	1.46	30.25	0.032
Stage	32.46	1.15	367.24	0.001

## Abbreviation

(CTCs): Circulating tumor cells
(NSCLC): Non-small-cell lung cancer
(Tregs): Regulatory T cells
(AJCC): American Joint Committee on Cancer
(PBMCs): Peripheral blood mononuclear cells
(SEM): Standard error of the mean.
